# Relationship between interRAI HC and the ICF: opportunity for operationalizing the ICF

**DOI:** 10.1186/1472-6963-9-47

**Published:** 2009-03-17

**Authors:** Katherine Berg, Harriet Finne-Soveri, Len Gray, Jean Claude Henrard, John Hirdes, Naoki Ikegami, Gunnar Ljunggren, John N Morris, Louis Paquay, Linda Resnik, Gary Teare

**Affiliations:** 1 Department of Physical Therapy, University of Toronto, Toronto, Canada

## Abstract

**Background:**

The International Classification of Functioning, Disability and Health (ICF) is embraced as a framework to conceptualize human functioning and disability. Health professionals choose measures to represent the domains of the framework. The ICF coding classification is an administrative system but multiple studies have linked diverse clinical assessments to ICF codes. InterRAI-HC (home care) is an assessment designed to assist planning of care for patients receiving home care. Examining the relationship between the ICF and the interRAI HC is of particular interest because the interRAI assessments are widely used in clinical practice and research, are computerized, and uploaded to databases that serve multiple purposes including public reporting of quality in Canada and internationally. The objective of this study was to examine the relationship between the interRAI HC (home care) assessment and the ICF. Specifically, the goal was to determine the proportion of interRAI HC items that can be linked to each of the major domains of the ICF (Body Function, Body Structure, Activities and Participation, and the Environmental Factors), the chapters and the specific ICF codes.

**Methods:**

Three coders who were familiar with both the home care assessment and the ICF independently assigned ICF codes to inter-RAI HC items. Subsequently, a series of teleconference meetings were held to reach consensus on the primary code and much later consensus was used to finalize codes for additional items added to the interRAI HC.

**Results:**

Following exclusion of administrative and diagnostic sections, 175 interRAI items were examined for potential assignment of codes. Of these 52 were assigned codes related to body function, 43 to activities and participation, 34 to environment, 1 to body structure, 17 to not coded, and 26 to not defined. Considering all 3-digit ICF codes, interRAI items addressed 43.2% of Body Function and 50.6% of Activities and Participation codes.

**Conclusion:**

The conceptual overlap in content, offers an excellent opportunity to operationalize the ICF domains and the codes particularly in the areas of Body Function and Activities and Participation. Use of measures such as the interRAI assessments with common elements across settings facilitates standardized reporting for organizations, regions and nations.

## Background

**Figure 1:**
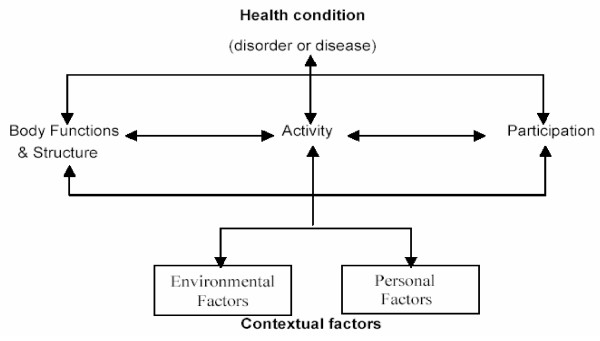
**Interactions between the components of ICF**. Source: WHO, 2001.

The concepts underlying the International Classification of Functioning, Disability and Health (ICF) are widely embraced by health professionals and educators. The framework, as shown in Figure 1, depicts an interrelationship among body structures and functions; activities and participation, and acknowledges relevant contextual personal or environmental factors that influence functional status and outcome. The framework encourages a broad view of the components of health for a patient beyond diagnosis and survival, and, as such, serves as a guide for comprehensive assessment. The popularity of the ICF framework encourages everyone to reflect on their assessment methods and measures to consider the relationship with the ICF.

The ICF belongs to the WHO family of classification systems which includes the International Classification of Disease (ICD), an international standard diagnostic classification for all general epidemiological and many health management purposes. Clinicians and researchers refer to the ICF framework in choosing measures to represent various domains [1] but there is no incentive to use the 1424 ICF codes in daily practice and indeed the ICF is a classification system rather than a measurement system. Cieza and colleagues [2,3] have developed rules to link health status measures to ICF codes. Iezzone and colleagues [4] have suggested that software be used to recode assessments used in daily practice to ICF codes in order to obtain common information on functional status in medical records without burdening clinicians or administrative staff with having to recode clinical assessments.

A key objective of the ICF is to improve communication among health professionals, administrators, governments and regions. This objective is shared by the interRAI "suite" of instruments which includes assessments for home care, post-acute care, palliative care, community health, long-term care and mental health. In contrast to the ICF classification, interRAI assessments have direct clinical utility for care and service planning, as well as having applications for outcome and quality monitoring and resource allocation. The assessment items, embedded scales, and quality indicators have been evaluated scientifically for reliability and validity [5-11]. Although there is variation in content among assessment instruments in the suite, they share common concepts and a common core of items that permit monitoring patient status across providers, sectors, and over time. This design feature is essential for an integrated information system.

The Home Care instrument, RAI-HC [12] is widely used in Canada and the US, and was the common assessment for an 11-country European study [13,14]. Other countries have rated the home care instrument as the best comprehensive assessment tool for use with individuals with chronic health conditions or disabilities (Australian Institute for Primary Care October, 2004; Belgian Institute for Insurance of Illness and Capacity 2003; 2006). The present study focused on an updated version (2006) of the RAI-HC, now called the interRAI HC, which is fully compatible with the suite of interRAI instruments. The interRAI HC is one of the longest assessments in the family. Its comprehensive nature creates an interesting opportunity to examine the relationship of the home care instrument to the ICF framework and coding classification. It would be an administrative benefit if the ICF could be generated from a computerized interRAI instrument.

The objective of this study, therefore, is to examine the relationship between the ICF and the interRAI HC. Specifically, the goal is to determine the proportion of interRAI HC items that can be linked to ICF framework domains (Body Function, Body Structure, Activities and Participation, and the Environmental factors), to their respective chapters and specific codes. This information would provide evidence of the conceptual relationship between the ICF classification and the interRAI assessments, and would assist in determining the feasibility of using interRAI assessments to generate ICF codes.

## Methods

Three individuals with expertise in geriatrics, rehabilitation, and performance measurement were assigned the task of linking the ICF codes to interRAI items. They were familiar with the interRAI instruments and with the ICF and reviewed both the WHO textbook on the International Classification of Functioning, Disability and Health and the methods for linking health status measures to the ICF codes developed by Cieza and colleagues [3]. The three coders independently assigned ICF codes to inter-RAI HC items. A research assistant assembled the codes into a table and distributed it to the three coders who were blinded to which codes were attributable to each of the other coders. After allowing several days for the coders to examine and reconsider their coding cross-walk in light of the information from the other coders a series of teleconference meetings were held to reach consensus on the optimal codes. Raters were asked to avoid the use of 'other specified' and 'other unspecified' ICF codes. The interRAI-HC items that related to concepts not addressed by the ICF codes were designated as "not covered" (NC) whereas items that could not be accurately linked to the most precise ICF code were designated as "not definable" (ND). The intention was to link interRAI items to a 3-digit code. If an item could not be linked to a 3-digit code but did fit to a chapter heading, it was assigned a chapter level code. Personal factors were recorded for descriptive purposes, with the understanding that they belonged within the framework but were not included in the coding structure as yet. Wherever possible, the coders documented difficulties and reasons for mismatches.

While this matching was being performed, there was ongoing development of the interRAI suite instruments prior to their release: certain items were dropped and others added. Coding of the new items was done by the lead author (KB) and submitted for verification to the original coders and to an outside expert in ICF coding. It was also decided that reporting of the ICF codes be limited to the primary code which was the one to which future qualifiers would be attributed. This was in contrast to the Cieza et al methodology [3] which suggested that all possible ICF concepts be linked when developing crosswalks to health status measures.

We examined the distribution and type of ICF codes linked to the interRAI items as well as the number of interRAI items that linked to each ICF chapter.

### Instrumentation

#### ICF

The ICF can be a companion to the ICD but is not intended for use only to describe deficits in function associated with disease. In contrast to the ICD which assigns codes when a disease or condition is present, the ICF permits the coding of both positive and negative features. All codes are written in neutral language, permitting consideration of deficits and strengths, barriers and facilitators. In total, there are 1,424 ICF codes within 30 chapters and four sections: Body Functions, Body Structures, Activities and Participation, and Environmental Factors, The codes are comprehensive, but there is no expectation that all areas for any one individual or group of individuals will be documented. "Core sets" have been identified that are condition-specific [15-18] and setting-specific [19,20].

The classification is represented by an alphanumeric coding system with the letter "b," "s," "d," and "e" referring to body function, body structure, activities and participation, and environmental factors respectively. These initial letters are followed by a numeric code that starts with the chapter number followed by 3 digits and 4 digit codes.

Whereas the neutral language of the ICF codes permits consideration of strengths and weaknesses, barriers and facilitators, there is a downside. It is not sufficient to simply list a code; there is a need to apply qualifiers to denote the extent or severity of a problem. The general qualifiers for three components are: no problem, mild problem, moderate problem, severe problem, complete problem, not specified, and not applicable. The Activities and Participation codes can be scored separately for performance and capacity, and the codes for environmental factors can be considered as facilitators or barriers. Reliability studies of the qualifiers have demonstrated low to moderate agreement [21-23]. There is potential for measurement error when assigning ICF codes and qualifiers from diverse clinical instruments.

#### The interRAI HC

The interRAI home care assessment, like the other members of the interRAI family, is a multi-dimensional assessment that addresses potential problem areas faced by individuals receiving home care. The original development of the instrument was an international endeavor with extensive input from clinical experts and organizations [12]. The assessment domains include cognition, psycho-social well-being, mood, behavior, physical function and activity, continence, pain, vision and hearing, nutritional status, skin condition, lifestyle, social support, preventive health, current diagnoses, treatments, and health service use. Combinations or single items signal a potential problem that may require attention in the care or service plan. The coding for items is based on objective, clear criteria, which has facilitated the reliability as assessed in multiple studies with independent paired ratings in various settings and countries [5,10,24,25]. The observation period for performance and observable behaviors is three days unless otherwise specified. There are core items that are found in all instruments and are defined the same way in each in order to encourage a common language and to facilitate their use in an integrated health system. The RAI Home Care/interRAI HC is used in daily practice in 9 US states, 8 Canadian provinces and numerous international settings as well as research projects including a 11 country home care project funded by the EU [13,14,26].

## Results

The initial sections of the interRAI home care assessment include identification numbers, reason for assessment and reference date for the assessment as well as other items referring to personal factors for which the ICF has no codes. Therefore, we excluded the identification, intake and initial history, and other administrative sections of the instrument. We also excluded 23 diagnostic items that were more appropriately linked to the ICD and the 14 items that related to treatments and procedures such as oxygen therapy and ventilator use that were most probably covered by coding procedure manuals. In total, 175 interRAI Home Care items were examined for potential linkage to ICF codes. The three raters, operating independently and without discussion, demonstrated agreement on 46% of the items. They reached consensus on the remaining items in teleconference discussions.

**Figure 2:**
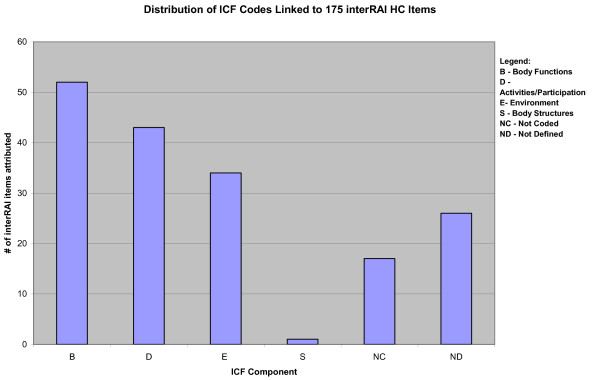
**Distribution of ICF codes assigned to 175 interRAI HC items**.

Figure 2 shows the distribution of ICF codes assigned to interRAI items. In total, 52 of the interRAI HC items were linked to ICF "B" codes denoting Body Function, 43 items were assigned to "D" codes for Activities and Participation, 34 to "E" codes for Environmental factors, and only one item an "S" code for Body Structure. An additional 17 interRAI HC items were considered not covered (NC) by the ICF and 26 items were labeled as ND-not precisely definable.

All items in the interRAI HC must be assessed for each home care patient. In contrast, ICF codes are not intended to apply or rather to be used for each individual. Nonetheless, it was of interest to examine the degree to which the interRAI HC addressed the ICF chapters, domains and codes. In the domain of Body Structure, there was only one 3-digit code linked to the home care instrument. As regards the Body Function domain, interRAI HC assessment addresses 43.2% of the 74 ICF 3-digit codes (excluding non-specific/other). Calculation functions were not coded by the interRAI HC but they are subsumed in the evaluation of the instrumental daily living task, managing finances. Items related to functional vision and hearing are included in the interRAI assessment but not other sensory functions such as taste and smell. The home care assessment does not address voice, fluency or articulation functions. Within Activities and Participation, 50.6% of ICF codes were addressed by the home care assessment. All codes within the Self-care chapter were addressed by the interRAI assessment. Codes not addressed included: transportation by animal, assisting others, caring for household objects and numerous items relating to preschool, school, apprenticeship and work.

The ICF codes and the interRAI HC include multiple codes or items relating to interpersonal relationships but there are different foci for each. Similarly in the Environmental Factors domain, there is a mismatch in specificity with regards to support and relationships. The ICF codes focus on type of relative providing support whereas the interRAI items focus on frequency of visits, and other interactions, regardless of type of relative. They include an element of time such as participation in activities of long-standing interest and change in social activities. In the area of social support and relationships, interRAI questions record the presence of primary caregiver feelings of distress, anger or depression and caregiver's inability to continue in caring activities. Although they include attitudinal aspects, the intent of the questions were to describe the availability of support and thus, were coded to the E3 chapter level. Similarly, the interRAI question of strong and supportive relationship with family (yes, no) was coded only to the E3 chapter level. Overall, only 12.7% of the Environmental Factors were covered by the interRAI HC.

Table 1 shows the number of items to which codes were attributed relative to each chapter of the ICF. The table underreports items that were conceptually linked to ICF but could not be assigned codes. For example, cognitive skills for decision making is assigned a code of D177 but no code was assigned to the item asking about a change in decision making in the past 3 months. Conceptually, the item is related to D1 but there was no possibility of coding a change in status. Similarly, other items addressing change in physical function and acute change in mental status (an indicator of delirium) could not be assigned specific codes.

**Table 1:** Number of interRAI items linked to ICF chapters

**ICF domains**	**Assigned codes**
**BODILY FUNCTIONS**	

Ch 1: Mental Functions	25

Ch 2: Sensory Functions and Pain	9

Ch 3: Voice and Speech Functions	0

Ch 4: Functions of the Cardiovascular, haematological, immunological and respiratory systems	5

Ch 5: Functions of the digestive, metabolic and endocrine systems	9

Ch 6: Genitourinary and reproductive functions	1

Ch 7: Neuromusc and movement-related functions	

Ch 8: Functions of the skin and related structures	5

**ACTIVITIES AND PARTICIPATION**	

Ch 1: Learning and applying knowledge	1

Ch 2: General tasks and demands	0

Ch 3: Communication	3

Ch 4: Mobility	12

Ch 5: Self-care	17

Ch 6: Domestic life	3

Ch 7: Interpersonal interactions and relationships	5

Ch 8: Major Life Areas	1

Ch 9: Community, social and civic life	1

**BODY STRUCTURES**	

Ch 1: Structures of the nervous system	

Ch 2: The eye, ear, and related structures	

Ch 3: Structures involved in voice and speech	1

Ch 4: Structures of cardiovasc, immuno and resp systems	

Ch 5: Structures related to digestive, metabolic, endocrine	

Ch 6: Structures related to genitourinary and reproductive	

Ch 7: Structures related to movement	

Ch 8: Skin and related Structures	

**ENVIRONMENTAL STRUCTURES**	

Ch 1: Products and technology	11

Ch 2: Natural + human-made changes to environment	1

Ch 3: Support and relationships	21

Ch 4:Attitudes	

Ch 5: Services, systems and policies	2

As noted on Table 1, the chapter with the largest number ICF codes linked to interRAI items was Body Function: Chapter 1- Mental Function. However, 13 of 14 mood items in the interRAI assessment linked to the same ICF code: B152, emotional function. Table 2 presents the 14 items and corresponding responses categories alongside the wording of the ICF item to which all were linked. Seven items in this section are summed to compute a Depression Rating Scale for used as an outcome measure A scoring of 3 or more out of 14 in the depression rating scale (DRS) is suggestive of depression and signals the need for care plan development and further investigation. Other interRAI items linked to Chapter B1 include symptoms of delirium, orientation, and memory. Despite covering only 12.7% of the 3-digit codes in Environmental Factors, 21interRAI items were attributed to the domain of Support and Relationships (E-Chapter 1). Activities and Participation were well covered by interRAI items, with 17 items linked to the Self Care domain and 12 to the Mobility domain. Moreover, the Self Care domain sub-headings were 100% covered.

**Table 2:** Example of crosswalk between the interRAI HC assessment and the ICF

**interRAI MOOD items**	**ICF**
	B 152 Emotional functions
**1. INDICATORS OF POSSIBLE DEPRESSION, ANXIETY, SAD MOOD**	Specific mental functions related to the feeling and affective components of the processes of the mind.
Code for indicators observed in past 3 days, irrespective of the assumed cause (note whenever possible, ask person)	
0 not present	
1 present but not exhibited in last 3 days	
2 Exhibited on 1–2 of last 3 days	
3 Exhibited daily in last 3 days	
a. Made negative statements e.g., "Nothing matters; Would rather be dead; What's the use; Regret having lived so long; Let me die"	Inclusions: functions of appropriateness of emotion, regulation and range of emotion, affect, sadness, happiness, love, fear, anger, hate, tension, anxiety, joy, sorrow, lability of emotion, flattening of affect.
b. Persistent anger with self or others e.g., easily annoyed, anger at care received	Exclusion: temperament and personality functions, energy and drive functions.
c. Expressions (including non-verbal) of what appear to be unrealistic fears e.g., fear of being abandoned, being left alone, being with others; or intense fear of specific objects or situations	Qualifier: 0. No problem 1. Mild problem 2. Moderate problem 3. Severe problem 4. Complete problem 8. Not specified 9. Not applicable
d. Repetitive health complaints e.g., persistently seeks medical attention, incessant concern with body functions	
e. Repetitive anxious complaints/concerns (non-health related) e.g., persistently seeks attention/reassurance regarding schedules, meals, laundry, clothing, relationships	
f. Sad, pained, or worried facial expression – e.g., furrowed brow, constant frowning	
g. Crying, tearfulness	
h. Recurrent statements that something terrible is about to happen – e.g., believes he or she is about to die, have a heart attack	
i. Withdrawal from activities of interest – e.g. long-standing activities, being with family/friends	
k. Expressions, including non-verbal, of a lack of pleasure in life (anhedonia) – e.g.	I don't enjoy anything anymore

**2. SELF-REPORTED MOOD**	
0 not in last 3 days	
1. not in last 3 days but often feels that way	
2. in 1–2 of the last 3 days	
3 daily in last 3 days	
8 person could not (would not) respond	
Ask: "in the last 3 days, how often have you felt..."	
a. Little interest or pleasure in things you normally enjoy	
b. Anxious, restless, uneasy	
c. Sad, depressed, hopeless	

Of the 130 items coded, fifteen were coded only to the chapter level. Three interRAI items – Timed Walk, Distance Walked and Distance Wheeled – each linked to Mobility, but were not defined beyond D4, the chapter level because they addressed additional dimensions that would influence any future qualification. These mobility dimensions of speed and distance are not captured by the ICF. Theoretically, future research could determine cut-points for speed and distance that correspond to ICF qualifiers but they would vary by population and setting.

Twelve items were coded to E3, the environmental chapter related to social support and relationships. As mentioned above, there was a mismatch of specificity between the ICF codes and interRAI items. The ICF were very specific as regards the type of relative. For other concepts, the ICF coding is not specific enough, for example in classifying contact with health professionals. In the ICF, health care professionals are grouped and classified by three codes (e340, e355, e360). The interRAI HC has separate items for key professionals in the home care setting.

Although we tried hard to avoid use of 'other specified' and 'other unspecified' ICF codes, the interRAI communication item relating to making self understood over the past 3 days does not specify method of communication whereas the ICF codes distinguish between verbal, non-verbal and other methods. Thus, it was only linked to the "other unspecified" code of D349.

**Figure 3:**
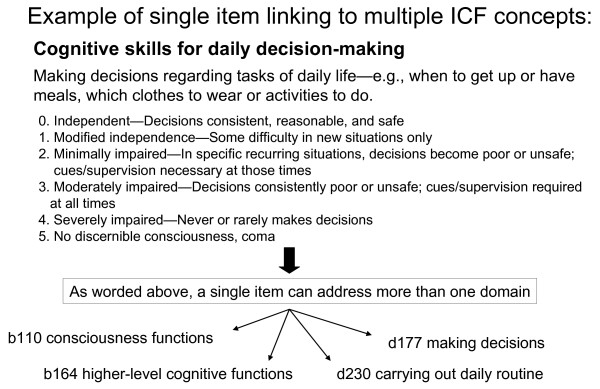
**Example of single item linking to multiple ICF concepts**.

There were challenges choosing the optimal ICF code for each item because interRAI items are defined relative to observable behaviours and functions and thus naturally link to multiple dimensions or concepts. For example, daily decision making, a core interRAI item can be linked to b110 consciousness functions, b164 higher level cognitive function, d 230 carrying out daily routine and d177 decision making. The optimal code chosen was d177 as this is the one which would be most meaningful when graded. (see Figure 3).

Several interRAI items had no corresponding concept or code in the ICF. For example, there was no representation of the construct of balance and no code available either for falls or for prior falls (a key predictor of falls). A history of pressure ulcers is also not included despite the fact that both history of falls and prior pressure ulcers place individuals at higher risk of future events in those areas. Similarly, recent change in mental status, decision-making, social activities and physical function are important markers of new problems and are predictive of outcomes. Wandering, a behavior associated with Alzheimer's, was also coded as NC. Other items not covered by the ICF include self-rated health and whether the person believes that he can improve in function, both of which would be considered personal factors. As yet, ICF codes for personal factors do not exist.

## Discussion

The present study has demonstrated that the interRAI HC content relates well to the ICF conceptual framework and that substantial numbers of items fall within the domains of Body Function, Activities and Participation, and Environmental Factors. The ability to assign ICF codes to instruments in daily practice is one way to propagate the use of the ICF and achieve the goals set forth by the WHO in developing this classification system. Multiple instruments have been linked to ICF codes [3,27], but none of the previously linked instruments have been as comprehensive an assessment covering many of the ICF domains or as widely used.

There are two practical applications of our demonstration that there is an interrelationship between the ICF and interRAI HC. First, jurisdictions or organizations that currently use the interRAI instruments but who wish to report ICF codes for monitoring health status may chose to apply the codes to the interRAI instruments without the need for a parallel system of administrative coding. Second, organizations or clinicians may wish to use an interRAI instrument to represent the framework of the ICF without assigning specific codes. This situation would parallel clinical use where clinicians and researchers choose multiple measures to represent the framework.

The idea of converting commonly used assessments to an international classification system for comparison is an attractive idea. However, it is important to note that how questions are asked or the method of assessment influence responses or scores [28]. For example, self reports of difficulty with daily tasks will yield higher rates of ADL limitations than questions pertaining to degree of assistance required with basic activities of daily living [29]. Thus, it is important to consider original items and methods of assessment when doing comparisons based on ICF codes derived from different types of measures. Similarly, comparisons based on scales with different underlying assessment methods should be interpreted with caution. Jurisdictions or organizations that use multiple interRAI instruments and sub-scales will have the benefit of communicating in the same language across settings and eliminating measurement error that arises from different methods of assessment.

Jurisdictions have adopted interRAI assessments for multiple purposes, including resource allocation and funding, informing policy decisions based on the persons' characteristics and outcomes, monitoring and improving quality of services, and public accountability. Despite these important administrative applications, the primary purpose of the assessments is care/service planning and clinicians are encouraged to use the subscales and quality indicators to monitor outcomes and quality indicators at a clinical level. Completion of the assessments is intended as an essential aspect of routine clinical practice rather than an added burden. The concurrent use of the instrument by clinicians for care planning and monitoring of outcomes enhances the accuracy of the data needed by administrators and the policy makers. The WHO statement that " the road leading to health for all passes through information" has been further elaborated to include: "a system of health statistics should both capture the current state of theory in the health domain and provide a framework for health information [30]. Such a health information template can serve as a pedagogical device, a comprehensive classification system, and a means to highlight gaps in our current range of data and collection systems" [30]. Given the substantial set of core items that cross the different interRAI family of assessments, there is a tremendous opportunity to use these instruments for standardized reporting.

The reliability of the interRAI assessments has been facilitated by use of functional and behavioral items to represent constructs. There are specific descriptors associated with each response option, facilitating the understanding of the extent of the limitations and the strengths of the person, and the services provided. A training manual addresses intent, and definition, and provides examples for scoring items. The detailed descriptions of the items and the scoring instructions have contributed to the reliability of the instruments [6,7,10]. This contrasts with ICF response coding options which are generic and rather vague (e.g., coding differentiates "mild," "moderate," and "severe" impairment, with a plus or minus sign to denote a positive or a negative situation). The reliability of the qualifiers or gradations of the ICF have been reported as below acceptable levels for clinical practice or research [22]. The ICF codes may be able to detect the presence of any problem in the population, but finer discrimination is essential when considering groups known to have limitations in functioning. As yet there is no evidence that ICF codes will be capable to form the basis of quality indicators or resource allocation.

Even if the primary focus of an intervention targets aspects of Body Function, the ICF framework reminds clinicians and researchers to measure the benefits in terms of activities and participation and to consider the influence of environmental factors. The ICF framework is not intended to be addressed fully by any single assessment instrument nor is it necessary that any individual be scored relative to each ICF code. There are now several recommended Core Sets for different patient populations. It was beyond the scope of this study to compare content of the Core sets and the interRAI HC instrument. Many of the ICF Core Sets have approximately one third devoted to body structure. In contrast, interRAI assessments focus on functional implications such that impairment to the eye would be captured based on its influence on a person's functional vision.

If a person is identified as needing an interRAI HC, all items must be completed. The focus is on function and behavior. Once problem areas or risk of problems are identified, health professionals are encouraged to do more in-depth assessment in that area. It can be argued that ICF codes not covered by InterRAI items are not applicable to all persons requiring home care. For example, mobility items referring to riding animals for transportation or kicking a ball do not seem essential. Another key difference between the approaches is the explicit decision not to focus on risk factors in the ICF classification. Clinical decision making is, however, largely based on prognostic factors that signal the need for care or service planning. The interRAI instruments have focused on comprehensive assessment with a specific emphasis on identifying potential problems that require further assessment or treatment.

Electronic health record (EHR) can be seen as a core data set of the most relevant administrative, demographic, and clinical information facts about a person's health care. There are multiple purposes for such information, including sharing of information across settings and among professionals, and longitudinal monitoring and maintenance of health records by individuals. Longitudinal monitoring, in particular, requires reliable information to permit detection of clinically important information even if it is of small magnitude. Coding classifications such as the ICF are unlikely to detect small true changes in status especially if different assessments are used. Although it is theoretically possible to convert the interRAI instruments and other reliable, valid measures to ICF codes, valuable information may be lost. E-health records should retain the original measures to increase precision in identifying risk and assessing small but meaningful change over time. This caveat is also true for other applications that may arise from electronic records such as quality indicators. It is essential that administrators have reliable data on which to make evidence based policy decisions. Health professionals who have embraced reliable and valid outcome measures over the past 20–30 years should be cautious to distinguish their support for the ICF framework from potential implications for the re-coding of measures with good measurement properties into ICF codes. For example, gait speed a highly recommended indicator of mobility would be recoded to the coarse qualifier: no problem, mild problem, etc.

The discord between the very specific ICF categories and the behaviorally structured interRAI questions created challenges and were likely responsible for the relatively low 46% level of agreement on the first round. For example, decisions had to be made as to whether calculation was a function that could be assigned a code because the Instrumental Activity of Daily Living (IADL) item, managing finances would presume the ability to calculate. Similarly, would the item Cognitive Skills for Daily Decision Making be only attributed to D177 or would it also cover higher thought functions.

A limitation of the current study is that we undertook the conceptual linkage to ICF codes but did not attempt to provide meaning to the codes by determining the appropriate qualifier codes. As previously noted, the qualifier codes add meaning to the neutral wording of the ICF codes. InterRAI responses grade severity specific to the domain. Self care is graded based on level of independence in performance whereas behaviours are graded based on the frequency in the past 3 days. Each would have to be converted to the generic ICF coding. In situations where multiple interRAI items link to a single ICF code, empirical analyses could examine the optimal combination of responses to determine the appropriate qualifier. For example, tobacco and alcohol use, taking medications, exercise and preventive health activities may be explored in combination to represent D570- looking after one's health. For local or national reporting, existing interRAI scales can be explored to represent ICF domains.

It is challenging to conclusively state the degree to which the interRAI (HC) covers the ICF. The method for assigning codes in this study focused solely on those items for which there was a reasonable expectation that a qualifier could be assigned. Other studies have advocated linkage to all possible concepts [2,3]. However, it is not clear as to how they would attribute the qualifiers and hence the meaning to the codes. Overall, the large number of interRAI items to which codes were assigned and the substantial coverage of the domains of Body function and Activities and Participation suggest that there is substantial conceptual overlap. Arguably, many of the ICF codes not covered may represent areas that are too specific for a comprehensive assessment or for national or international reporting.

## Conclusion

In summary, our objective to demonstrate compatibility between the ICF and the interRAI HC systems was primarily met. While assigning codes and achieving agreement between the coders was not straight forward, a consensus was reached. Future work would be required to convert interRAI response codes to ICF qualifier codes. Health professionals involved with interRAI assessments can be reassured that they cover substantial domains of the ICF, particularly in the domains of Activities/Participation and Body Function. The ability to cross-walk items and scales within the RAI-HC assessment to the ICF framework shows great potential for the systems to co-exist and complement each other to serve the common purpose of standardizing functional status information.

## Competing interests

The authors declare that they have no competing interests.

## Authors' contributions

KB was involved in the conceptualization, writing and analysis of the manuscript. All authors contributed to writing of the manuscript and critically revised the drafts of the paper. LR, KB and GT participated as raters, and were involved in editing of the manuscript. All authors read and approved the final manuscript.

## Pre-publication history

The pre-publication history for this paper can be accessed here:



## References

[B1] Barak S, Duncan PW (2006). Issues in selecting outcome measures to assess functional recovery after stroke. NeuroRx.

[B2] Cieza A, Geyh S, Chatterji S, Kostanjsek N, Ustun TB, Stucki G (2005). ICF linking rules: an update based on lessons learned. J Rehabil Med.

[B3] Cieza A, Brockow T, Ewert T, Amman E, Kollerits B, Chatterji S, Ustun TB, Stucki G (2002). Linking health-status measurements to the international classification of functioning, disability and health. J Rehabil Med.

[B4] Iezzoni LI, Greenberg MS (2003). Capturing and classifying functional status information in administrative databases. Health Care Financ Rev.

[B5] Hawes C, Morris JN, Phillips CD, Mor V, Fries BE, Nonemaker S (1995). Reliability estimates for the Minimum Data Set for nursing home resident assessment and care screening (MDS). Gerontologist.

[B6] Hirdes JP, Poss JW, Curtin-Telegdi N (2008). The Method for Assigning Priority Levels (MAPLe): a new decision-support system for allocating home care resources. BMC Med.

[B7] Morris JN, Fries BE, Mehr DR, Hawes C, Phillips CD, Mor V, Lipsitz LA (1994). MDS Cognitive Performance Scale. J Gerontol.

[B8] Morris JN, Nonemaker S, Murphy K, Hawes C, Fries BE, Mor V, Phillips C (1997). A commitment to change: revision of HCFA's RAI. J Am Geriatr Soc.

[B9] Morris JN, Fries BE, Morris SA (1999). Scaling ADLs within the MDS. J Gerontol A Biol Sci Med Sci.

[B10] Mor V, Angelelli J, Jones R, Roy J, Moore T, Morris J (2003). Inter-rater reliability of nursing home quality indicators in the U.S. BMC Health Serv Res.

[B11] Berg K, Mor V, Morris J, Murphy KM, Moore T, Harris Y (2002). Identification and evaluation of existing nursing homes quality indicators. Health Care Financ Rev.

[B12] Morris JN, Fries BE, Keel K, Ikegami N, Bernabei R, Carpenter GI, Gilgen R, Hirdes JP, Topinkova E (1997). Compresensive clinical assessment in community setting: applicability of the MDS-HC. J Am Geriatr Soc.

[B13] Carpenter I, Gambassi G, Topinkova E, Schroll M, Finne-Soveri H, Henrard JC, Garms-Homolova V, Jonsson P, Frijters D, Ljunggren G, Sørbye LW, Wagner C, Onder G, Pedone C, Bernabei R (2004). Community care in Europe. The Aged in Home Care project (AdHOC). Aging Clin Exp Res.

[B14] Bos JT, Frijters DH, Wagner C, Carpenter GI, Finne-Soveri H, Topinkova E, Garms-Homolova V, Henrard JC, Jonsson PV, Sorbye L, Ljunggren G, Schroll M, Gambassi G, Bernabei R (2007). Variations in quality of Home Care between sites across Europe, as measured by Home Care Quality Indicators. Aging Clin Exp Res.

[B15] Brach M, Cieza A, Stucki G, Fussl M, Cole A, Ellerin B, Fialka-Moser V, Kostanjsek N, Melvin J (2004). ICF Core Sets for breast cancer. J Rehabil Med.

[B16] Ewert GS (2004). How to asses the impact of arthritis on the individual patient: The WHO and ICF. Annals of the rheumatic diseases.

[B17] Cieza A, Ewert T, Ustun TB, Chatterji S, Kostanjsek N, Stucki G (2004). Development of ICF Core Sets for patients with chronic conditions. J Rehabil Med.

[B18] Stucki G, Cieza A (2004). The International Classification of Functioning, Disability and Health (ICF) Core Sets for rheumatoid arthritis: a way to specify functioning. Ann Rheum Dis.

[B19] Grill E, Hermes R, Swoboda W, Uzarewicz C, Kostanjsek N, Stucki G (2005). ICF Core Set for geriatric patients in early post-acute rehabilitation facilities. Disabil Rehabil.

[B20] Stoll T, Brach M, Huber EO, Scheuringer M, Schwarzkopf SR, Konstanjsek N, Stucki G (2005). ICF Core Set for patients with musculoskeletal conditions in the acute hospital. Disabil Rehabil.

[B21] Okochi J, Utsunomiya S, Takahashi T (2005). Health measurement using the ICF: test-retest reliability study of ICF codes and qualifiers in geriatric care. Health Qual Life Outcomes.

[B22] Grill E, Mansmann U, Cieza A, Stucki G (2007). Assessing observer agreement when describing and classifying functioning with the International Classification of Functioning, Disability and Health. J Rehabil Med.

[B23] Uhlig T, Lillemo S, Moe RH, Stamm T, Cieza A, Boonen A, Mowinckel P, Kvien TK, Stucki G (2007). Reliability of the ICF Core Set for rheumatoid arthritis. Ann Rheum Dis.

[B24] Hirdes JP, Ljunggren G, Morris JN, Frijters D, Finne-Soveri H, Gray L, Bjorkgren M, Gilgen R (2008). Reliability of the interRAI suite of assessment instruments: A 12-country study of an integrated health information system. BMC Health Services Research.

[B25] Gray L, Bernabei R, Berg K, Finne-Soveri H, Fries BE, Hirdes JP, Jonsson P, Morris J, Steel K, Arino-Blasco S (2008). Standardising assessment of elderly people in acute care: the interRAI Acute Care instrument. J Am Geriatr Soc.

[B26] Henrard JC, Ankri J, Frijiters D, Carpenter I, Topinkova E, Garms-Homolova V, Finne-Soveri H, Sorbye Jonsson PJ, Ljunggren G, Schroll M, Wagner C, Bernabei R (2006). Proposal of a service delivery integration index of home care for older persons: application in several European cities. Int J Integr Care.

[B27] Mayo NE, Poissant L, Ahmed S, Finch L, Higgins J, Salbach NM, Soicher J, Jaglal S (2004). Incorporating the International Classification of Functioning, Disability, and Health (ICF) into an electronic health record to create indicators of function: proof of concept using the SF-12. J Am Med Inform Assoc.

[B28] Walsh EG, Khatutsky G (2007). Mode of Administration Effects on Disability Measures in a Sample of Frail Beneficiaries. Gerontologist.

[B29] Laditka SB, Jenkins CL (2001). Difficulty or dependency? Effects of measurement scales on disability prevalence among older Americans. J Health Soc Policy.

[B30] Wolfson MC, Evans RG, Barer ML, Marmor TR (1994). Social Proprioception: Measurement, Data, and Information from a population Health perspective. Why are some people healthy and others not?.

